# Sport Mega-Events and Displacement of Host Community Residents: A Systematic Review

**DOI:** 10.3389/fspor.2021.805567

**Published:** 2022-01-07

**Authors:** Claudio M. Rocha, Zixuan Xiao

**Affiliations:** Faculty of Health Sciences and Sport, Sport Management Programme, University of Stirling, Stirling, United Kingdom

**Keywords:** sustainable development goals, sport mega-events, evictions, removals, Olympic Games, gentrification

## Abstract

The aim of this study was to conduct a systematic literature review to understand how empirical data have informed the knowledge about the relationship between hosting sport mega-events and displacement of host community residents. Following the PRISMA protocol, we conducted a search of academic and gray literature in sport, social sciences, and humanities databases. We excluded conceptual papers, conference abstracts, and works that discuss urban transformation or displacement but are not related to sport events. We also excluded works that associate sport mega-events with urban transformations but are not related to resident displacement. From the initial 2,372 works reviewed, 22 met the inclusion criteria. In empirical studies, displacement of residents has been studied exclusively in the context of the Olympic Games, since Seoul 1988, but with a higher frequency in most recent Games (Beijing, London, and Rio). The gigantism and the sense of urgency created by the Olympic Games may explain why this event has been frequently associated with resident displacement. Findings showed that residents suffered either direct, forced evictions or indirect displacements. The selected studies show a contradiction between the discourse of sport mega-events guardians for supporting the United Nations Sustainable Goals (SDG) and the practice of human rights within host cities of such events.

## Introduction

The beginning of the relationship between the International Olympic Committee (IOC) and the United Nations (UN) can be dated back in the 1920s, when there were some discussions between Pierre de Coubertin and the League of the Nations (“La Société des Nations”), a forerunner of the UN (Grosset and Attali, 2008). After the foundation of the UN in 1945, discussions progressed mainly through the United Nations Educational, Scientific and Cultural Organization (UNESCO). In 1952, UNECO's executive board authorized the organization to be represented at the Olympic Games in Helsinki (Meier, 2017). Since then, the organizations have gotten closer. In 2009, the IOC became a Permanent Observer of the UN, an honor usually reserved for non-member states, but very rarely granted to non-governmental organizations (Van Luijk, 2018). In 2017, the UN and the IOC agreed on a direct partnership. According to the IOC, this direct partnership “will help sport to fulfill its role as “an important enabler of sustainable development”, as outlined in the United Nations Sustainable Development Goals” (IOC, 2017). Controversially, this direct partnership led to the closure of the UN Office on Sport for Development and Peace (Burnett, 2017). The “Fédération Internationale de Football Association” (FIFA) has also been considered a partner of the UN. In 1999, FIFA and the UN signed a strategic alliance to promote education through football (Sadecky, 2006). In 2021, the UN Deputy Secretary-General, Ms. Amina Mohammed said that, “The United Nations welcomes the collaboration with FIFA, particularly football's potential in supporting the SDGs” (FIFA, 2021).

Despite formal and informal partnerships between the UN and the guardians of sport mega-events, such events have been associated with abuses of human rights, especially related to displacement of host community residents (Boykoff, 2014; Lenskyj, 2016; Williamson, 2017; Horne, 2018). The 2015 Human Rights Watch annual report identified the five most critical human rights abuses associated with sport mega-events and listed evictions and displacements of citizens as the number one violation (Horne, 2018). There is an estimation that more than two million people were removed from their houses by the Olympic gentrification between Seoul 1988 and London 2012 Games (COHRE, 2007; Monks, 2016). Reports and conceptual papers have described that forced evictions or displacements of residents have happened in all host cities of sport mega-events in the last 30 years (Vale and Gray, 2013; Boykoff, 2014; Williamson, 2017). This fact seems to indicate the lack of commitment that the guardians of sport mega-events have had with the UN Sustainable Development Goal (SDG) 11–Make cities and human settlements inclusive, safe, resilient, and sustainable.

In this research, we use Grier and Grier's (1978, p. 8) definition of displacement, where a displacement occurs when “household are forced to move from its residence by conditions that affect the dwelling or its immediate surroundings, and that: (a) are beyond the household's reasonable ability to control or prevent, (b) occur despite the household's having met all previously imposed conditions of occupancy, and (c) make continued occupancy by that household impossible, hazardous, or unaffordable” (Grier and Grier, 1978). Marcuse (1985) notes that this definition encompasses all forms of displacements, including direct and indirect displacement. Eviction is a term that is commonly used to denote forced, direct, and/or involuntary displacement (Easton et al., 2020). In our systematic search, we consider both terms–displacement and eviction–to prevent missing important works. In the context of sport mega-events, displacement has been described as a consequence of gentrification (Watt, 2013; Richmond and Garmany, 2016; Easton et al., 2020). Pearman (2018, p. 127) defines gentrification as “a type of physical, economic, and cultural transition in low-income urban neighborhoods in which disinvested, oftentimes minority neighborhoods subsequently experience an influx of wealthier households and increases in real property values”. We also looked for academic works that associate gentrification with sport mega-events to approach not only direct, but also indirect displacement.

For the current study, we use Müller's (2015, p. 638) definition of mega-events, which are defined as “one-off events of a fixed duration that attract a large number of visitors, have a large mediated reach, come with large costs, and have large social and environmental impacts”. Muller builds upon previous definitions of mega-events (Roche, 1994; Hiller, 1999; Horne, 2007; Gold and Gold, 2016) to identify four key factors that separate mega-events from other events: tourist attraction, media reach, costs, and host place transformation. The literature has placed an emphasis on the Olympic Games and the FIFA World Cup as examples of sport mega-events. However, other sporting events can also be considered mega-events, depending on their characteristics related to the key factors (Müller, 2015).

In the literature, we have a mix of books, book chapters, conceptual papers, and opinion articles about the association between sport mega-events and displacement of residents (Lenskyj and Wagg, 2012; Boykoff, 2014; Horne, 2018). In gray literature, we have media reports describing the size of the problem (Kelso, 2004; Comitê_Popular_do_Rio, 2013; Assadourian, 2014). Knowledge about the topic has grown a lot in the last decade. However, that remains a fragmented knowledge, which creates difficulties for researchers and policy makers to know the state-of-the-art of the field. The literature review as a research methodology is acknowledged as an important tool to tackle this problem (Snyder, 2019). For a literature review to work effectively as a research methodology it has to be accurate, precise, and trustworthy (Snyder, 2019). Scholars have described systematic literature reviews as an effective research methodology because this type of review seeks to systematically search for, appraise and synthesize previous works (Grant and Booth, 2009; Moher et al., 2015). Systematic literature review has been suggested over other types of literature reviews in areas where research is fragmented and interdisciplinary and when researchers look to inform policy and practice (Snyder, 2019). Therefore, take into consideration the characteristics of research about sport mega-events and displacement and the aim of this study, we opted for conducting a systematic literature review.

We are unaware of any other published systematic literature review on sport mega-events and displacement of residents in local communities. We do not know how much of the knowledge produced so far has been produced after empirical studies and what information such studies can collectively bring to the field. The lack of a systematic review limits our knowledge about the current state-of-the-art of the topic. This knowledge is fundamental to inform both the need of future empirical studies on the topic and the action of decision makers. It can also inform about theoretical frameworks and methodologies that have been used to investigate the problem and the ones that are missing. Therefore, the aim of this study was to conduct a systematic literature review to understand how empirical data have informed the knowledge about the relationship between hosting sport mega-events and displacement of host community residents.

## Materials and Methods

**Figure 1:**
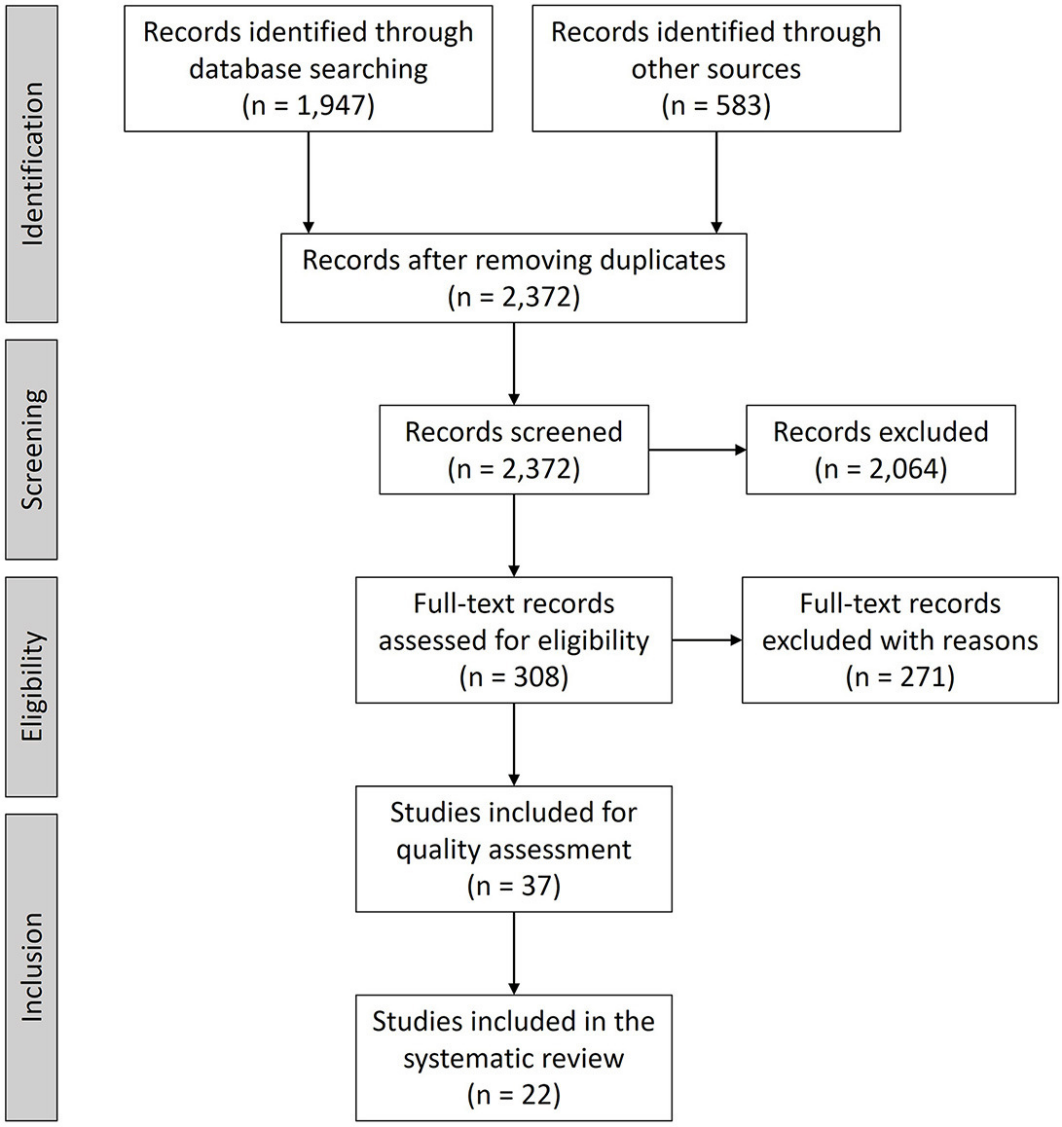
PRISMA flow diagram.

This systematic review was conducted following the PRISMA protocol, following Moher et al.'s (2015) guidelines. Figure 1 provides the PRISMA flow chart. First, as a research team, we discussed and listed all terms that are associated with impacts of hosting sport mega-events on local resident displacement. Then, we searched for the terms in sport and broader social and humanities academic data bases (WebScience, Scopus, Sport Discus, Proquest, SocIndex, Public Affairs Index, and Political Science Complete) and gray literature and theses [Google Scholar, Open Access Theses and Dissertations (OATD) and Open Gray]. Next, we downloaded all references from the initial search results and imported them into Mendeley, which allowed us to identify and remove all duplicates.

### Search Strategy

To ensure the broadest capture of publications possible, we used the following search terms, in different locations of the files, such as TI = title, AB = abstract, KW = keyword, and SU = subject. Below, we list the terms and locations.

[*TI* (displacement^*^ OR eviction^*^ OR gentrification OR urban renewal OR neighbo#rhood change OR demolition^*^ OR urban regeneration OR urban transformation) OR *AB* (displacement^*^ OR eviction^*^ OR gentrification OR urban renewal OR neighbo#rhood change OR demolition^*^ OR urban regeneration OR urban transformation) OR *KW* (displacement^*^ OR eviction^*^ OR gentrification OR urban renewal OR neighbo#rhood change OR demolition^*^ OR urban regeneration OR urban transformation) OR *SU* (displacement^*^ OR eviction^*^ OR gentrification OR urban renewal OR neighbo#rhood change OR demolition^*^ OR urban regeneration OR urban transformation)]

*AND*

[*TI* (sport^*^ event^*^ OR sport^*^ mega event^*^ OR sport^*^ major event^*^ OR Olympic^*^ OR World Cup) OR *AB* (sport^*^ event^*^ OR sport^*^ mega event^*^ OR sport^*^ major event^*^ OR Olympic^*^ OR World Cup) OR *KW* (sport^*^ event^*^ OR sport^*^ mega event^*^ OR sport^*^ major event^*^ OR Olympic^*^ OR World Cup) OR *SU* (sport^*^ event^*^ OR sport^*^ mega event^*^ OR sport^*^ major event^*^ OR Olympic^*^ OR World Cup)]

### Study Selection

In the search, we found 2,530 records (1,947 records from database searching and 583 from other sources–see Figure 1). We uploaded the list into Mendeley and removed the duplicates. After eliminating duplicates, 2,372 records were found. The next steps were conducted independently by both authors. After each step, we met to discuss and resolve divergences. To screen the records, we started by checking the title and the abstract of the records. A set of pre-selected inclusion and exclusion criteria were used throughout the screening process that was designed to capture as many outlets as possible. The inclusion criteria were Journal articles, academic books, academic book chapters, theses, gray literature, and empirical studies written in English. They should have included the search terms in title, keywords, abstract, or subject. The exclusion criteria were: Conceptual papers, conference abstracts, works that discuss urban transformation, gentrification or displacement but are not related to sport events, and works that associate sport mega-events with urban transformation or gentrification but are not related to resident displacement.

After the screening step, we excluded 2,064 records and finished with 308 full-text records to be assessed for eligibility. After reading the full text and applying the exclusion criteria, we eliminated 271 records with reasons and kept 37 records for quality assessment.

### Quality Assessment

To assess the quality of the 37 remaining records for final inclusion, analysis, and synthesis, we followed previous systematic reviews in sport management (Parent and Hoye, 2018) and used the American Psychology Association's guidelines (Mallinckrodt, 2011). The quality assessment resulted in 15 records being excluded due to problems with the application of methods, lack of scientific contribution to the problem of displacement of local community residents, or lack of focus on displacement of residents. The final collection of the systematic review included 22 empirical papers.

### Data Extraction and Analysis

For each of the 22 records included in the final collection of the systematic review, we extracted the following information: source (journal, university or institution), authors and date, event (location and year), research aim, theoretical framework, method, main findings, and main limitations. Each author extracted the information individually and, later, met to resolve divergences. We focused on identifying strengths, similarities, and differences among the final records. The extracted data allowed us to discuss four main points: (1) the type of events (including location and year) that have been associated with resident displacement, (2) the theoretical frameworks used to study the topic, (3) the research methods, and (4) the main findings (existence or not of displacement in the context of sport mega-events, type of displacement, strategies used to displace, and type of population affected).

## Results

Table 1 summarizes the 22 studies included in the systematic review. Sixteen studies were articles published in peer-reviewed journals, while four were theses and two, reports. The studies were published between 2006 and 2020, with 18 of them (82%) being published between 2012 and 2020. Only four studies were conducted before 2012.

**Table 1:** Summary of extracted data from studies included in the systematic review.

**Source**	**References**	**Event**	**Research aim**	**Theoretical framework**	**Method**	**Findings**	**Limitations**
Planning Perspectives	Bernstock, 2020	Olympic Games–London 2012	To interrogate four key policy assumptions that underpinned the strategy for delivering affordable housing in the context of London 2012 Games.	The Growth dependent Planning Paradigm. Neoliberalism.	Document analysis.	Hosting London 2012 the revalorization of land stimulated private sector investment. Despite a rhetoric of inclusion, a neoliberal model was applied coupled with planning gain. This is a relatively weak instrument for redistributing value/benefits to disadvantaged communities. New housing developments are aimed at more affluent populations, contributing to gentrification and displacement of poor residents.	Use of document analysis is the only source of information. Single case study.
Bournemouth University	Cotton, 2018	Olympic Games–London 2012	To understand how the urban regeneration caused by London 2012 Olympic Games affected the daily lives of residents in the host area.	Neoliberalism.	Walking ethnography (observation), photovoice methodology (using Instagram), and semi-structured interviews (*n* = 4)	Within post-Olympic East London, the findings reveal the ubiquitous processes of neoliberal urbanization and gentrification which has contributed to the marginalization of poor residents, in opposition to new middle-class incomers. Poor residents have been displaced to peripheral hinterlands.	Single case study. Limited number of participants. All male participants. Association between findings and theory with room for improvement.
International Journal of Urban and Regional Research	Davis, 2011	Olympic Games–Seoul 1988	To assess the effect of hosting the 1988 Seoul Olympic Games on the city's housing situation, evictions record, and social movement groups.	No theoretical framework. Descriptive research.	Document analysis.	There was a marked increase in housing demolitions, eviction, dislocation, and new units of housing built in the period of Olympic Games preparations. The practice of eviction did become more frequent and more violent as a direct result of Olympic Games preparations. There were traces of an emerging housing rights movement which gains influence after the Olympic Game.	Use of document analysis is the only source of information. Single case study.
Urban Geography	Gaffney, 2016	Olympic Games–Rio 2016	To examine different cases in the context of pre-Rio 2016 Olympic Games to determine whether gentrification is occurring and, if so, by what processes.	No theoretical framework. Descriptive research.	Document analysis.	In Rio, sport mega-events have catalyzed and consolidated stages of urban change with particularly acute reflections in the real estate sector. In 2016, a multiplicity of gentrifications were happening. Gentrification has involved the displacement of poor residents by a wealthier group that exhibits different cultural patterns.	Limited use of documents. Lack of theoretical framework.
Environment and Planning D: Society and Space	Gillespie et al., 2018	Olympic Games–London 2012	To reflect on the austerity urbanism imposed by the London 2012 Olympic Games on single mothers after the destruction of social housing in post-Olympics East London.	Austerity urbanism framework. Marxist feminism.	Participant observation. Semi-structured interviews (*n* = 12).	The Carpenter's Estate occupation demonstrates the gendered nature of the urban commons and the leadership of women in defending them from enclosure. The process of commoning provided the basis for the forging of enduring relationships, the strengthening of existing networks and the circulation of ideas, creating an Olympic “counter-legacy” that exists in opposition to the legacy of the 2012 Games.	Single case study.
Housing, Theory and Society	Humphry, 2020	Olympic Games–London 2012	To explore how social tenants' lived experiences in post-London 2012 Olympic East Village are shaped by new forms of neoliberalism embedded into housing provision.	Individualization theory.	Semi-structured interviews (*n* = 32). Participant observation.	After London 2012, in the East Village, there has been a shift from patterns of residualization to individualization, as self-reliant tenants are sought above those most in housing need. Social housing discourse seems to be shifting from notions of need to concerns with affordability.	Single case study. Unique context.
Urban Studies	Kavetsos, 2012	Olympic Games–London 2012	To estimate the impact of the London 2012 Olympics announcement on property prices.	No theoretical framework. Econometric model.	Secondary data from MousePrice and from the Land Registry (the official recording authority for property transactions in the UK). Hedonic model of the selling price of housing in host areas tested via regression analysis.	It is estimated that properties in host boroughs are sold between 2.1 and 3.3 per cent higher and properties up to three miles away from the main Olympic stadium are sold for 5 per cent higher. It is likely that inflationary pressures on the price of land and property will displace the poorest individuals currently residing in East London.	Lack of theoretical framework. Data driven investigation. Lack of further discussion about social impacts.
Sociology	Kennelly and Watt, 2011	Winter Olympic Games–Vancouver 2010; Olympic Games–London 2012	To assess the experiences of homeless youth in light of claims made by Vancouver 2010 and London 2012 organizers that the Games would benefit young people in host cities.	Spatial Dimensions of Global Spectacles (Lefebvrian spatial analysis along with Debord's concept of spectacle).	Interviews and focus groups in Vancouver (*n* = 27) and London (*n* = 10).	Disproportionate policing to “clean the streets” was a major issue experienced by homeless youth in Vancouver. There are indications that the same was happening in London. The Olympic Games are not equivalent to other gentrification and urban cleansing efforts. Their status as “mega-events” contribute to unexpected spatial results for homeless youth.	Exploratory nature of interviews in London.
Visual Studies	Kennelly and Watt, 2012	Olympic Games–London 2012	To examine the impact of the London 2012 Olympics on low-income and marginally housed young people living in the London borough of Newham (host area of the Games).	No theoretical framework. Descriptive research.	Photo elicitation interviews (*n* = 13).	The Stratford area of Newham (near to the Olympic Park) has visibly changed and increasingly threatened to exclude marginalized youth. Indirect displacement of youth happened in the area of Newham as a consequence of Olympic gentrification.	Lack of theoretical framework. Data driven investigation. Single case study.
COHRE	Mahon, 2007	Olympic Games–London 2012	To explore the preparations underway to host the 2012 Olympic Games and report the impacts on housing rights that were already becoming evident in the East London host area.	No theoretical framework. Descriptive research.	Document analysis.	Five years before the Games, over 1,000 people faced the threat of displacement from their homes, and housing prices were escalating. These effects were being disproportionately felt by marginalized groups: the poor, low-income earners, residents of public housing, and ethnic minorities such as Romani Gypsies and Irish travelers.	Exploratory in nature. Lack of theoretical framework. Single case study.
University of British Columbia	Pentifallo, 2015	Winter Olympic Games–Vancouver 2010	To critically examine the City of Vancouver's urban policies during the lead up to the 2010 Winter Olympic and Paralympic Games.	Critical Urban Theory.	Document analysis. Critical policy analysis.	The 2010 Games brought an intense wave of punitive urban measures that functioned to criminalize homelessness and validate the removal of Vancouver's homeless population. Additionally, the number of social housing units made available shrunk, eventually dwindling to a minimal number of units actually constituting social housing.	Use of secondary data only. Document analysis is the only source of information.
Pennsylvania State University	Rock, 2012	Olympic Games–Beijing 2008	To investigate how low-income and marginalized center city residents navigate the new political, economic and socio-cultural terrain to resist urban dispossession and dislocation, pre- and post-Olympics in Beijing.	Neoliberalism.	Participant observation. Survey question-naires (n = 86). Document analysis.	The displacement and relocation of Beijing's hutong residents exemplifies a different kind of negotiated sacrifice and burden. There is no residential choice that can prevent the demolition of their hutong homes. People were displaced from “hutong” neighborhoods to high-rise formations, cleared of the previous history. Former hutong residents living in public housing received little, if any, compensation for being removed from their homes.	Use of “informal” interviews. Small sample size for questionnaires.
Bournemouth University	Sadd, 2012	Olympic Games–London 2012	To develop a framework of urban regeneration legacy associated with the hosting of mega-events. To critically analyse Olympic social legacy with particular reference to the long-term positive, soft benefits.	Stakeholder theory	Semi-structured interviews (n = 22).	In the context of the Olympic Games, planned urban regeneration can easily become an example of urban gentrification if no protection is given to the local “working class” population. The local community to the site of the Games, whether they are residents, businesses, societies, clubs or communities, must be identified and consulted to achieve any form of long-term sustainable positive social legacy outcomes.	Problems of trying to repeat the same individuals in each case study (cases are far apart in time).
COHRE	Sanchez et al., 2007	Olympic Games–Barcelona 1992	To assess the possible relationship between the preparations for the Barcelona 1992 Olympic Games and the practice of forced evictions. To investigate the impact of the event on the enjoyment of the right to adequate housing in the city.	No theoretical framework. Descriptive research.	Document analysis. Interviews.	The urban development that started with the Olympic Games had negative consequences such as degradation of traditional neighborhood and displacement of residents. The construction of the Olympic village removed 147 families, mainly elderly widows and low-income families. Hosting the Games had a negative impact on housing affordability in the city, illustrated by strong increases in the prices of housing for rent (+145%) and for sale (+139%).	Exploratory in nature. Lack of theoretical framework. Single case study.
Environment & Urbanization	Shin and Li, 2013	Olympic Games–Beijing 2008	To critically examine the experience of migrant tenants, local tenants, and landlords in villages in the city (Cheongzhong-cun) in regard to their experiences on the preparations for the 2008 Olympic Games.	No theoretical framework. Descriptive research.	Semi-structured interviews (n = 48)	Immediately after Beijing 2008, migrants suffered eviction and displacement after demolitions. They receive neither cash nor in-kind compensation, which is available only for local permanent residents. Migrants faced exclusionary experiences of the Olympic Games, although they were main workforce for the Olympic city construction.	Exploratory in nature. Lack of theoretical framework. Single case study.
Leisure Studies	Suzuki et al., 2018	Olympic Games–Tokyo 2020	To address and explain forced evictions that have taken place due to the reconstruction of the National Stadium in Tokyo to host the 2020 Olympic Games.	No theoretical framework. Descriptive research.	Semi-structured interviews (*n* = 15). Survey questionnaire (*n* = 43)	The reconstruction of the National Stadium for the 2020 Olympics induced displacement of two groups of vulnerable people: homeless people and tenants of a public housing estate.	Lack of theoretical framework. Data driven investigation. Exploratory in nature. Small sample size for questionnaires.
Leisure Studies	Talbot and Carter, 2018	Olympic Games–Rio 2016	To examine activists' use of human rights as a discourse to contest the impacts of the Rio 2016 Olympic Games.	No theoretical framework. Descriptive research.	Participant observation. Ethnographic research.	Activists fighting against forced evictions explicitly used the language of human rights in their activism. The media accounts tended not to discuss these issues using this lexicon. The issue of police violence was considered an abuse of human rights much more regularly than that of eviction, suggesting that there exists a hierarchy of rights, with some more respected than others.	Lack of theoretical framework. Data driven investigation. Exploratory in nature.
Habitat International	Wang et al., 2015	Olympic Games–Beijing 2008	To analyse the relationship between mega-event regeneration and expected residential relocation outcomes.	Gentrification and displacement framework. Econometric model.	Survey questionnaire (*n* = 880).	State-sponsored regeneration leads to landscape improvement and social upgrade yet is often accompanied by residential displacement and social marginalization. Residents who lived in the area affected by the Olympics and have a lower level of education and/or income are more likely to face displacement caused by the Olympic Games.	The causal link between expectation and decision in the context of housing was not tested. Lack of discussion on measures to alleviate or eliminate displacement of poor residents in the context of Olympic gentrification.
City	Watt, 2013	Olympic Games–London 2012	To examine the 2012 Olympic Games legacy in relation to the displacement experiences of lower-income East Londoners.	No theoretical framework. Descriptive research.	Participant observation. Ethnographic research.	The East London residents in deprived areas learned that the Olympic gentrification scheme in Newham was not occurring for their benefit. Displacement processes could well mean that they would no longer be able to live in their current neighborhood. The Olympics legacy is for others, not for them.	Lack of theoretical framework. Exploratory in nature.
Cities	Watt, 2018	Olympic Games–London 2012	To assess how gender, housing, austerity, and the right to the city inter-relate with reference to female lone parents from East London, the site of the 2012 Olympic Games.	Right to the city framework	Semi-structured interviews (n = 12). Participant observation.	The post-Olympics' legacy has largely failed to address the housing needs of East London's low-income population. The lone parents could not afford the accelerating private rents or house prices in post-Olympics' East London and understandably prefer to access the diminishing pool of social rental housing which is still relatively secure and affordable. Their capacity to do so has, however, been reduced. Women's right to the city has eroded.	Single case study. Exploratory in nature.
Leisure Studies	Yoon, 2020	Winter Olympic Games–Pyeong-Chang (2018)	To explore the experiences of residents who lived near Mount Gariwang, a protected area that was developed into an alpine ski venue; and to examine how locals may have felt compelled to consent to the development.	Post-politics framework	Semi-structured interviews (*n* = 12).	Post-politics processes have contributed to obtaining consent for the destruction of an ancient forest on Mount Gariwang. The process of dispossession and displacement was not entirely coercive, but rather, transpired via manufactured consent.	Single case study. Demographic homogeneity of interviewees. Not all interviews were recorded.
Real Estate Economics	Zheng and Kahn, 2013	Olympic Games–Beijing 2008	To examine the gentrification consequences of public investments in Beijing, the host of the 2008 Olympic Games.	No theoretical framework. Econometric model.	Secondary data from the National Bureau of Statistics of China. Multiple regression analysis.	The city government invested in the Olympic Village and in new subways, leading to increases in local home prices, construction, and restaurants of higher quality nearby. Higher-income and more highly educated households are attracted to live in those places. These investments have caused local gentrification. Homes of poor people, such as rural migrants, are demolished and they are pushed further out to the remote suburban areas.	Lack of theoretical framework. Data driven investigation. Lack of discussion about social impacts. Exploratory in nature.

Regarding the type of event, all studies that made the final collection were conducted in the context of the Olympic Games. Most of the studies approached the association between the Summer Games and displacement. Only three studies had the Winter Games as the context (Kennelly and Watt, 2011; Pentifallo, 2015; Yoon, 2020). Eleven studies were conducted to investigate the case of London 2012, while four were about the case of Beijing 2008, two were about the case of Rio 2016 and other two, about Vancouver 2010. Almost all works investigated a single Olympic event. There is only one exception (Kennelly and Watt, 2011) where a comparison between London 2012 and Vancouver 2010 was conducted. Beyond the studies that are part of the final collection of this systematic review, there are some reports that compared hosts, such as the COHRE report (COHRE, 2007).

The studies have drawn upon a variety of theoretical frameworks. While there is no dominance of a specific theory, theories related to the use of urban space–the right to city framework (Lefebvre, 1996), theory of spatiality (Lefebvre, 1991), and neoliberalism (Harvey, 2005)–were discussed in some articles (Rock, 2012; Cotton, 2018; Watt, 2018; Bernstock, 2020). An important finding is that 50% of the studies have not drawn upon any theory or theoretical framework. These are studies that have a descriptive nature, aiming to investigate whether displacements in host cities of sport mega-events have happened and discussing some possible causes and consequences. Three studies developed econometric models, representing a group of articles with similar aims of estimating the impact of hosting sport mega-events on housing factors (e.g., construction, rent, and house prices), which have been impacting displacement of residents (Kavetsos, 2012; Zheng and Kahn, 2013; Wang et al., 2015).

Most of the studies apply the single case study approach, using qualitative strategies to analyse data extracted from either interviews (10 studies) or documents (nine studies) or observations (seven studies). Among studies that used qualitative data analysis, six applied more than one source of information, combining interviews with observations and/or document analysis. In contrast, only two studies relied on survey questionnaires to collect data, using quantitative strategies to analyse data (Wang et al., 2015; Suzuki et al., 2018). One of these two studies used both quantitative and qualitative data analysis techniques, with a quite small sample size for survey questionnaires. The other one collected data from a large sample and used quantitative data analysis techniques (Wang et al., 2015). An additional study collected data using questionnaires, but analyzed the data qualitatively (Rock, 2012).

Regarding the main findings, studies that are included in the systematic review have an overall message that preparing to host sport mega-events, more specifically the Olympic Games, has led to displacement of host community residents. In all 22 studies, findings showed that residents suffered either direct or indirect displacement or both. Direct displacement has included eviction of marginalized residents as consequence of preparing to host events such as Seoul 1988, Barcelona 1992, Beijing 2008, Vancouver 2010, London 2012, Rio 2016, and PyeongChang 2018 Olympic Games (Watt, 2013; Zheng and Kahn, 2013; Pentifallo, 2015; Talbot and Carter, 2018). Most of the studies found that indirect displacement of marginalized groups happens due to gentrification, revalorization of land, and negative impact on housing affordability. Marginalized groups include the poor, low-income earners, social housing residents, elderly, homeless people, youth, and ethnic minorities (Mahon, 2007; Kennelly and Watt, 2011; Gillespie et al., 2018; Yoon, 2020). In general, studies investigated eviction and displacement of marginalized residents from houses. Few studies approached the displacement of special populations such as homeless people of host cites (Kennelly and Watt, 2011; Pentifallo, 2015; Suzuki et al., 2018).

## Discussion

The aim of this study was to conduct a systematic literature review to understand how empirical data have informed the knowledge about the relationship between hosting sport mega-events and displacement of host community residents. There is an agreement among the studies that preparing to host sport mega-events has led to displacement of marginalized groups of residents. Strategies to displace residents have varied between forced evictions and indirect displacements. Government of host cites have used different tactics to apply such strategies in different ways and proportions. An analysis of the sport mega-events that appear in the final collection of the systematic review reveals some similarities and differences in the use of displacement tactics.

The context of all studies is the Olympic Games, mainly the Summer Games. Findings show that displacement of residents has been an issue at least since Seoul 1988. Analyzing document data, Davis (2011) found that around 48,000 buildings were removed between 1983 and 1988, displacing 720,000 residents in densely populated, low income districts of Seoul (Davis, 2011). Davis asserted that official documents support a causal link between forced evictions and preparations for the 1988 Games. Davis (2011) affirmed that the Seoul city hall openly used the 1988 Games (and the 1986 Asian Games) to promote a “city beautification,” which necessarily included demolition of old buildings and removal of poor communities. The study reveals that both forced evictions and city beautification led to a massive displacement of poor residents of Seoul in preparation for the Games. The numbers make the Seoul 1988 the sport mega-event that led to second largest catalyst of resident evictions, only behind to Beijing 2008 (Vale and Gray, 2013).

In a different context from Seoul, Barcelona 1992 led to some evictions, but it was essentially about indirect displacement of residents. Sanchez et al. (2007) investigated how urban development initiated by preparing the city for the Games affected house affordability and, consequently, displacement of economically disadvantaged residents (Sanchez et al., 2007). Sanchez et al. reported that 147 families were removed for the construction of the Olympic village, mainly elderly, widows, and low-income families. They also found that hosting the Games had a negative impact on affordability housing in the city, illustrated by strong increases in the prices of housing for rent (+145%) and for sale (+139%) in the period between 1986 and 1993. This was one of the first studies to conclude that the Olympic Games have served to exacerbate the consequences of privatization of housing, contradicting the human rights perspective where housing is seen as a basic need (Sanchez et al., 2007).

In the systematic review, we found a gap of empirical studies between Barcelona 1992 and Beijing 2008. We found in the literature, two studies that relied on comparisons between the cases of Atlanta 1996, Sydney 2000, Athens 2004, and other events (Kontokosta, 2012; Teeuwen, 2018) to discuss rights to housing. These studies were two of the 37 studies included for quality assessment. They were not included in the final collection of the systematic review because they are not directly related to resident displacement. But they offer some important contribution to understand housing issues in the context of those sport mega-events between Barcelona and Beijing. They echoed some of the problems that were reported in other settings–mainly increase in the prices of houses and rents, which can lead to indirect displacement of specific marginalized groups in the host city.

Beijing 2008 inaugurated a new era in the studies about sport mega-events and displacement. For the first time, studies started to use explicitly the framework of neoliberalism to explain Olympic gentrification and displacement of residents in a socialist host country (Harvey, 2003, 2005; Rock, 2012). Beijing 2008 was the scenario of four empirical investigations about displacement of residents (Rock, 2012; Shin and Li, 2013; Zheng and Kahn, 2013; Wang et al., 2015). Overall, the studies reported a state-sponsored gentrification, which led to evictions and indirect displacement of marginalized groups (e.g., residents with lower levels of education and/or income). Three of those studies investigated displacement of poor residents in the hosting area. For instance, Zheng and Kahn (2013) found that the gentrified area close to the Olympic Village attracted people with higher incomes and higher levels of formal education, pushing poor residents to farther remote suburban areas. Shin and Li (2013) investigated the effects of hosting on a very specific group of residents–the migrants (Shin and Li, 2013). They found that after the Beijing 2008, migrants had their houses demolished and received neither cash nor in-kind compensation. They were likely to be the most marginalized group of residents in the city. Beyond forced evictions, all studies in Beijing reveal a scenario of gentrification in the host area where poor residents could no longer afford living there. The selected empirical studies support some conceptual papers that have been written about the case of Beijing 2008 and displacement of residents (Broudehoux, 2007; Shin, 2013; Ren, 2017). There is an estimate that 1.25 million people were displaced from their houses in Beijing as consequence of hosting the 2008 Games (Vale and Gray, 2013).

Displacement has been rarely investigated in the context of Winter Olympic Games. The Winter Olympics have been less investigated than the Summer Olympics in all aspects (Chappelet and Kübler-Mabbott, 2008). In this systematic review, we found three exceptions. Two of them approached the Vancouver 2010 Games (Kennelly and Watt, 2011; Pentifallo, 2015). Kennelly and Watt (2011) and Pentifallo (2015) focused on the problem of displacement of homeless people from the streets, symbolizing an attempt to “clean” the city of Vancouver to receive the Games. Some conceptual works about the Vancouver 2010 case are aligned with the findings of the empirical articles (Vanwynsberghe et al., 2013; Boykoff, 2014). The Vancouver Games have been associated with punitive urban measures that criminalized homelessness and validated displacement of residents (Pentifallo, 2015). This specific marginalized group investigated in the case of Vancouver has similarities with other groups such as migrants in the context of Beijing 2008 (Shin and Li, 2013) and female lone parents in London 2012 (Watt, 2018). Still in the context of Winter Olympics, one study approached the case of PyeongChang 2018 (Yoon, 2020). Yoon (2020) found that elderly people living in an environmentally protected area in Mount Gariwang “agreed” to leave for the construction of the alpine ski venue for the 2018 Games. That “agreement” happened after local authorities applied post-politic tactics to convince them that their displacement was serving to a greater good.

With 11 empirical studies, London 2012 has been by far the most investigated Games regarding displacement of residents (Mahon, 2007; Kennelly and Watt, 2011, 2012; Kavetsos, 2012; Sadd, 2012; Watt, 2013, 2018; Cotton, 2018; Gillespie et al., 2018; Bernstock, 2020; Humphry, 2020). Some studies were conducted before the Games and others, after. Comparing these studies, we can see that previsions of displacement before the Games were confirmed by facts after the Games. Mahon (2007) reported that 5 years before the Games, over 1,000 people was facing threats of evictions from their homes, while housing prices were escalating, increasing chances of indirect displacement. Findings after the Games showed that marginalized groups were either evicted by demolition of their houses or indirectly displaced by gentrification (Watt, 2013; Cotton, 2018; Bernstock, 2020). In a later study, Watt (2013) reported that 400 houses were demolished at the Clays Lane estate in Newham, East London (Watt, 2013). Most of the studies, however, explored the relationship between preparing to host the Games and indirect displacement *via* gentrification. Bernstock (2020) and Cotton (2018) found that the 2012 Games led to the gentrification of the host area. They agree that the city government applied a neoliberal model to justify the gentrification of the area. The neoliberal model applied in London mirrors that used in Beijing some years before. The gentrified area in East London attracted new middle-class residents, displacing poor residents from the hosting area to other areas of the city.

Rio 2016 seems to be the apotheosis of the use of neoliberalism to gentrify areas and displace marginalized residents, mainly in the informal settlements of city called favelas. The literature has many conceptual papers about the problem of favela evictions in preparation for Rio 2016 Olympic Games (Richmond and Garmany, 2016; Sanchez et al., 2016; Ivester, 2017; Williamson, 2017). There is congruence between such papers and the two empirical articles that made the final selection of this systematic review. Talbot and Carter (2018) investigated the case of Vila Autódromo, a favela located a few meters from the Olympic Park (Talbot and Carter, 2018). They found that preparations to host led to activist resistance. While Vila Autódromo activist resistance became a case of success reported around the world (Ivester, 2017; Williamson, 2017), it did not, unfortunately, avoid the almost total removal of the community, along with other human rights abuses such as police violence (Talbot and Carter, 2018). Gaffney (2016) added that Olympic gentrification affected different areas of the city in different ways (Gaffney, 2016). However, in favelas, gentrification has unleashed market forces, which residents cannot cope with, just having the option to move out to other localities, characterizing indirect displacement. Gaffney's findings support some conceptual papers (Vannuchi and Van Criekingen, 2015; Freeman and Burgos, 2017) that proposed that neoliberalism has led to gentrification and displacement in Rio. A dossier prepared by an independent agency revealed that 77,206 residents were displaced to make way for Olympic infrastructure in Rio (Robertson, 2015; Boykoff, 2017).

Displacement of marginalized populations has been also reported in the context of Tokyo 2020. Suzuki et al. (2018) found that the reconstruction of the National Stadium for the 2020 Games led to displacement of two vulnerable groups: homeless people and tenants of a public housing estate (Suzuki et al., 2018). Similarly to what happened in Barcelona (Sanchez et al., 2007) and Vancouver (Kennelly and Watt, 2011), the case of Tokyo does not show mass eviction (as it happened in Beijing and Rio). Rather, it shows that specific, highly vulnerable groups were affected by authorities' decisions to change the structure of the city. Such decisions have caused both evictions and indirect displacements not only in Tokyo but also in London and Vancouver.

The absence of studies in the context of other sport mega-events is an important finding of the systematic review. The Olympic Games have special characteristics that have made them likely to promote resident displacement and, consequently, have attracted the attention of researchers. We discuss more about this in the conclusions. Two studies included for quality assessment were conducted in the context of FIFA World Cup. These were not included in the final collection of the systematic review because they were not specific about resident displacement from their houses. Ohmann et al. (2006) explored the perceived social impacts of the 2006 FIFA World Cup on Munich residents. They reported that one of the social impacts was that residents did not perceive an increase in house rents or prices, making displacement unlikely in Munich. Maharaj (2017) investigated the displacement of subsistence fisher folk from their workplace, during the regeneration of the port of Durban in preparation for the 2010 FIFA World Cup (Maharaj, 2017). Maharaj found that fisher folk were denied access to their workplace (Durban's beachfront) to make the area attractive for international tourists. Such fishers engaged in public protests with little effect.

The presence of different theoretical frameworks in the selected studies revealed that the problem of displacement can be analyzed from different points of view. Three studies applied econometric models, to use to use real-world data to explain what was happening in the housing market (Kavetsos, 2012; Zheng and Kahn, 2013; Wang et al., 2015). Two of these studies were essentially descriptive, informing that rent and house prices had increased in the host area leading to displacement of marginalized communities. The exception was Wang et al. (2015) who tested an econometric model based on Freeman and Braconi's (2004) framework (Freeman and Braconi, 2004), where displacement was a function of residents' demographics and gentrification (social and physical regeneration). Wang et al.'s results supported the theory, informing that Beijing residents with lower levels of education and/or income were more likely to be displaced from areas affected by Olympic gentrification. The relationship between demographics that characterized marginalized populations and displacement is consistently found in other Olympic cities (Kennelly and Watt, 2011; Watt, 2013; Talbot and Carter, 2018; Yoon, 2020).

Theories related to the use of urban space–the right to city framework (Lefebvre, 1996), theory of spatiality (Lefebvre, 1991) and neoliberalism (Harvey, 2005)–have informed a group of studies that appear in the final collection of the systematic review (Kennelly and Watt, 2011; Cotton, 2018; Gillespie et al., 2018; Watt, 2018; Bernstock, 2020). The link between Lefebvre's theories of urban space (Lefebvre, 1991, 1996) and neoliberalism is made through the concept of “accumulation by dispossession” (Harvey, 2003). Harvey (2003) proposed that, when neoliberal capitalism cannot generate growth anymore, then a redistributive process called “accumulation by dispossession” is put into practice (Harvey, 2003). In this process, privatization of land and displacement of poor residents are necessary to transfer capital from the poor to the rich. Hosting Olympic Games has been very efficient to attain both–privatization of the land and displacement of poor. To feed capitalism, the state needs to increase the value of the land in poor areas of the city. Therefore, the sites of the Olympic venues seem not to have been chosen by chance. In recent years, in many instances, local governments have chosen deprived areas of cities to build Olympic venues–from London borough of Newham (Watt, 2013) to Jacarepaguá in Rio (Sanchez et al., 2016). The argument is that the presence of venues would improve infrastructure and make the lives of the residents in those areas better. This has been the discourse in Olympic bids. Ex post facto reality has shown that, instead of improving the lives of current residents, infrastructure improvement has created accumulation by dispossession, displacing such residents from their houses. Once residents are out, the land can be negotiated by higher prices. A group of studies in this systematic review followed the logic of accumulation by dispossession in their theoretical arguments, but they rarely mentioned it explicitly. Most of the studies in this group prefer to draw upon the concept of neoliberalism–a political approach that decreases the control of the state and increased free-market capitalism. Therefore, another important contribution of the systematic literature review was to point accumulation by dispossession as a strong theory to explain displacement of marginalized residents as consequence of hosting sport mega-events.

## Conclusions

Although we have conducted the systematic review looking for any type of sport mega-event, all empirical studies that made the final collection have investigated displacement of residents in the context of the Olympic Games. The unique characteristics of the modern Olympic Games may explain why this event has been associated with resident displacement. The Olympic Games is the world's largest sport event hosted by only one city^1^. The centralization of such a huge event has created an allegedly need for large infrastructure changes in the host city. Preparing to host the Games has demanded constructions and improvements not only in sport facilities, but also in the city infrastructure, including airports, ports, green areas, roads, and public transportation. This demand used to occur in a relatively short period of time (usually 7 years^2^). A demand for large infrastructure changes in a short period of time helped host city authorities and organizers to create a sense of urgency. Under the assumption of delivering the event with excellence and on time, authorities have promoted what some authors have called a “state of exception,” where business people and some local authorities are more likely to circumvent local and international laws, including human rights agreements, to attain the capital interest (Agamben, 2005; Vainer, 2011; Richmond and Garmany, 2016). Unfortunately, the state of exception in the context of the Olympic Games seems to have created a fertile terrain for neoliberal practices and displacement of residents. We are not aware of studies in the literature associating a state of exception with other sport events, maybe because they have not been so apparently urgent as the Olympic Games. With host cities now being selected earlier and not based on bids anymore, we hope that the argument of urgency will lose its appeal. The new approach to electing Olympic and Paralympic host cities takes an important step by pointing to the importance of existing facilities (IOC, 2021). The problem of being a gigantic event hosted in only one city is still there to be considered.

The systematic review revealed some limitations in the published empirical studies and possibilities of future studies. From a theoretical point of view, half of the empirical studies were conducted as descriptive investigations, with no theory supporting the research. This is a limitation of current studies. While descriptive studies may have their importance, the literature seems to have room to progress to more theory-based studies. One possibility for future studies is to expand the use of theories of urban space. For instance, the theoretical framework of accumulation by dispossession (Harvey, 2003) offers an intuitive possibility to inform future studies. Beyond theories of urban space, other theories can inform future studies. The stakeholder theory, which was used by one study in the systematic review (Sadd, 2012), can inform future studies by exploring the role of other important parties involved in the displacement process beyond the dyad local government-residents. These parties can be local stakeholders such as non-governmental organizations, public attorneys, public agencies, and private real estate organizations. They can also be international stakeholders, such as the IOC, the UN, and international human rights organizations.

From a methodological point of view, almost all studies (but two) that used primary data collection relied on qualitative methods to analyse data from documents and/or interviews. This reveals an emphasis on the interpretive paradigm. From the positive side, to date, studies have provided valuable in-depth data for us to understand the problem. But we have areas for improvement regarding quantitative studies. The studies that have been based on the positivist paradigm have analyzed secondary data to present econometric models (usually describing the effects of hosting on housing factors). But there is a lack of quantitative studies using primary data collection. Quantitative data collected from survey questionnaires, for example, should help to inform not only about special demographics of displaced residents, but also about their attitudes and behaviors when facing threats and/or actual displacements. Due to the nature of the problem, our suggestion is that mixed methods research should be beneficial to expand the knowledge. Qualitative studies are still necessary because we will need to understand the effects of new events (with their unique contexts) on displacement of residents. Quantitative studies could help to broaden our knowledge and explain, for example, whether and why residents resist evictions and displacements and whether and what strategies they use.

Regarding the context of the studies, more studies are necessary about the Winter Olympic Games. Beyond the three empirical studies that are in the final collection of this review (Kennelly and Watt, 2011; Pentifallo, 2015; Yoon, 2020), some studies have shown that the Winter Games host cities have applied neoliberal policies, as have the Summer Games (Vanwynsberghe et al., 2013; Müller and Gaffney, 2018). As a hypothesis, this might have led to indirect displacement of residents. But we have limited data to support this. Investigating the next Winter Olympics host cities–Beijing 2022 and Milan-Cortina 2026–may provide data to test that hypothesis. Beyond the Olympic Games, other sport mega-events can also be investigated. Some conceptual papers have discussed the problem of marginalized populations and displacement in the context of recent FIFA World Cup (Pillay and Bass, 2008; Kassens-Noor and Ladd, 2019). Empirical research is still in need. Other sport events, when they demand large infrastructural changes in the city, could also inform for future studies on the topic of displacement.

From the review, we missed studies investigating the role of the IOC, a key stakeholder in the process of hosting the Olympic Games. Most of the studies highlight only the responsibility of local governments for displacements. While the local government surely has much responsibility on this, the role of the IOC (and other sport mega-event guardians and governing bodies) should not be ignored. The IOC cannot control national policy in sovereign countries. But the IOC has certainly a role to play. The one responsible for granting host rights should not turn a blind eye to clear cases of human right abuses in host cities/countries of their most important event (Rocha et al., 2021). Acknowledging that resident evictions and displacements in host cities is also *their* problem can be the first step to show a practical support for the SDG 11–Make cities and human settlements inclusive, safe, resilient, and sustainable.

We conclude from the selected studies in the systematic review that a contradiction has existed between the discourse of sport mega-event guardians (offering full support to the UN SDG) and the practice adopted in preparing cities to host. In practice, those involved in the preparation of cities to host (local government, organizing committee, sport mega-event guardians) have shown little concern for the SDG 11. The first target of the SDG 11 reads, “By 2030, ensure access for all to adequate, safe and affordable housing and basic services and upgrade slums” (United_Nations, 2015). Results of the systematic review showed that hosting the Olympic Games has been directly linked to eviction and displacement of marginalized residents. Thus, hosting has neither promoted adequate, safe, and affordable housing to all, nor helped to improve lives in deprived areas. Our results support some conceptual papers that have pointed the contradiction between discourse and practices (Horne, 2018). Evictions and displacements of marginalized residents are listed as the number one abuse of human rights (Worden, 2015).

## Author Contributions

CR was responsible for the conceptualization and writing of the article. ZX was responsible for searching the records, uploading them into Mendeley and removing the duplicates. All other steps were conducted independently by both authors, who met regularly to resolve divergences.

## Funding

This work was supported by the University of Stirling Open Access and Article Processing Charge Fund.

## Conflict of Interest

The authors declare that the research was conducted in the absence of any commercial or financial relationships that could be construed as a potential conflict of interest.

## Publisher's Note

All claims expressed in this article are solely those of the authors and do not necessarily represent those of their affiliated organizations, or those of the publisher, the editors and the reviewers. Any product that may be evaluated in this article, or claim that may be made by its manufacturer, is not guaranteed or endorsed by the publisher.

## Footnotes

^1^Olympic competitions of some few sports (e.g., football/soccer) have happened in different cities of the same country. This is an exception. Almost all competitions are centralized in the host city.

^2^This has recently changed as the IOC granted the host rights of the 2028 Games to Los Angeles in 2017, and the 2032 Games to Brisbane in 2021 – this one using the new approach to electing Olympic and Paralympic host, not based on bids anymore (https://olympics.com/ioc/future-host-election).
